# Too Fat

**Published:** 1882-11

**Authors:** 


### TOO FAT.

THE tendency to accumulate fat is constitutional ; but it can be either checked or encouraged by the individual in whom this tendency exists. In spite of all protestations to the contrary, fat persons eat heartily ; they may not consume as much food as some who are lean, but they require less, because there is more complete assimilation ; that is, the nutrient portions of the food are more readily converted into fat, and there is less waste. All medicines that cause a reduction of fat, do so at the expense of the general health. Bottled anti-fat remedies are simply bottled disease, since it would be impossible to reduce the amount of adipose with one bottle or a hundred bottles of it, unless it was sufficiently powerful to derange the natural and healthful functions of the body and induce disease. It would be safer to accomplish the object by contracting fever and ague, or even small pox. There is a method, however, of reducing excessive fatness, that is not only safe but healthful ; and which will insure greater comfort and perhaps longer life to the individual.

The fat-producing foods are fats and those that contain starch or sugar ; and these are the foods that strong stomachs crave most.

Such foods should be used in moderation ; but we must not fall into the fatal error of discarding them altogether, as was years years ago suggested by Banting. Many of his followers exchanged obesity for fatal kidney diseases. It would be well for an overfleshy person to make a "bill of fare" for himself, on a descending scale as to non-nitrogenous foods, and an ascending scale as to nitrogenous foods. He should diminish the quantity of wheat bread eaten at each meal, gradually down to one or two ounces. The same for fats and potatoes. He should make up the deficiency with lean meats, and bread made from flour from which most of the starch had been extracted. The next most important points are regular out-door labor, and abstinence from all kinds of liquor and beer. Half a cup of tea or coffee at each meal, taken without milk or sugar, is allowable. Avoid drinking too freely of water. It is said water makes flesh, and this in one sense is true ; it causes the food to be more rapidly but less thoroughly digested ; and where water is in excess of the requirements of digestion, much of it is taken up by the absorbents and carried into the tissues of the body, adding to their bulk.

It must be borne in mind that excess of flesh is not always an indication of health. It may indicate disease of a serious nature. What has been said does not of course apply to cases of this kind. These require a far different treatment. Finally, any competent physician could prescribe a regimen for any individual case of obesity—where no marked disease exists—that would surely and safely accomplish its object, and thus save the patient from the perils of anti-fat preparations.

In the next number of the JOURNAL we shall discuss the opposite condition, under the title, "Too Lean."

